# Septic Arthritis of the Sternoclavicular Joint Complicated by Empyema and Bronchopleural Fistula: A Case Report

**DOI:** 10.7759/cureus.76358

**Published:** 2024-12-25

**Authors:** Yu Metsugi, Kikuo Shigemitsu, Tomoki Hirata, Yuma Iwamoto, Hideyoshi Sato

**Affiliations:** 1 Plastic and Reconstructive Surgery, Ogaki Municipal Hospital, Ogaki, JPN; 2 Thoracic Surgery, Ogaki Municipal Hospital, Ogaki, JPN

**Keywords:** bronchial occlusion, empyema necessitatis, endobronchial watanabe spigots, negative pressure wound therapy (npwt), pectoralis major muscle flap, pleural empyema, pyothorax, septic arthritis, sternoclavicular joint (scj) septic arthritis, sternoclavicular reconstruction

## Abstract

Septic arthritis of the sternoclavicular joint is a rare infectious arthritis in which the risk factors are reported to be such as diabetes, immunosuppression, and intravenous drug use. Due to a lack of prominent symptoms, delayed diagnosis can lead to severe complications such as mediastinitis and empyema. Advanced sternoclavicular septic arthritis can be a hidden etiology masked by severe symptoms. Therefore, awareness of this condition is crucial for timely and appropriate treatment. We report a rare case of sternoclavicular septic arthritis progressing to empyema with bronchial fistulae. The patient was successfully treated with multidisciplinary interventions, including endoscopic bronchial occlusion, open-window thoracostomy, negative-pressure wound therapy, sternoclavicular joint debridement, and reconstruction with a contralateral pectoralis major flap. This report discussed the treatment strategy with a review of the previous literature.

## Introduction

Septic arthritis of the sternoclavicular joint (SASCJ) accounts for approximately 1% of septic arthritis cases and is considered a rare condition [1,2]. Risk factors include intravenous drug abuse, diabetes, rheumatoid arthritis, and immunosuppression. *Staphylococcus aureus* is the most common causative organism (49-65%), and hematogenous spread is a possible route of infection [2-4]. Clinical symptoms primarily include pain around the sternoclavicular joint (SCJ) and fever, although isolated joint swelling may also occur.

Treatment options range widely, from radical debridement and reconstruction with musculocutaneous flaps, as recommended in some reports [2,5], to conservative treatment with antibiotics alone [6], depending on the clinical presentation. Herein, we report a rare case of septic sternoclavicular arthritis complicated by fistulous empyema that required multidisciplinary management, including endoscopic bronchial occlusion, open-window thoracostomy, SCJ debridement, and reconstruction with a pectoralis major muscle flap. A literature review is provided to discuss the treatment strategies for such advanced cases.

## Case presentation

The patient was a 68-year-old man who presented with a subcutaneous mass in the anterior chest region that had appeared four weeks prior. Two weeks before admission, he developed progressive anorexia, fever, and gait difficulty, prompting him to seek medical attention. His medical history included poorly controlled insulin-dependent diabetes mellitus. On admission, his vital signs included a temperature of 37°C, a heart rate of 100 bpm, and a blood pressure of 99/77 mmHg. Laboratory tests revealed elevated inflammatory markers (CRP: 23.9 mg/dL, WBC: 15,960/μL), hyperglycemia (random glucose: 627 mg/dL, HbA1c: 12.9%), and subcutaneous edema in both lower extremities.

**Figure 1:**
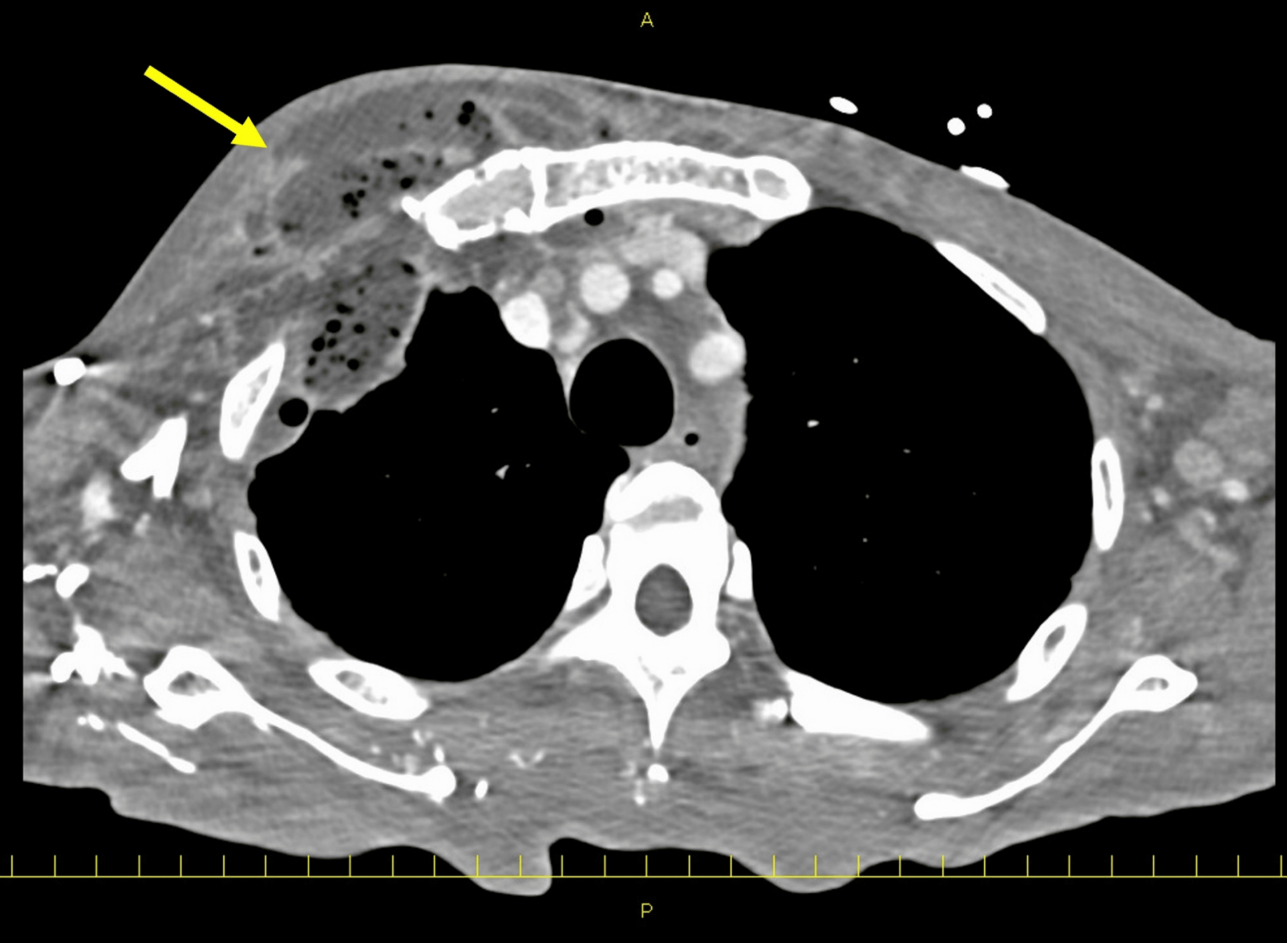
CT image at initial presentation An abscess (yellow arrow) was observed extending from the subcutaneous tissue of the right anterior chest to the thoracic cavity and the mediastinum behind the sternum. The first costal cartilage was dissolved and disconnected.

Contrast-enhanced CT showed abscess formation extending from the subcutaneous tissue of the anterior chest to the SCJ and chest wall, nodular shadows indicative of septic pulmonary embolism, and bilateral gluteal subcutaneous abscesses (Figure 1). The patient underwent surgical drainage by the thoracic surgery team on the day of admission, revealing purulent discharge from the subcutaneous tissue, first intercostal space, and pleural cavity. A bronchopleural fistula was also identified, leading to a diagnosis of fistulous empyema, for which an open-window thoracostomy was performed. The second rib was resected approximately 3 cm, and the infected, dehisced first sternocostal joint was removed.

**Figure 2:**
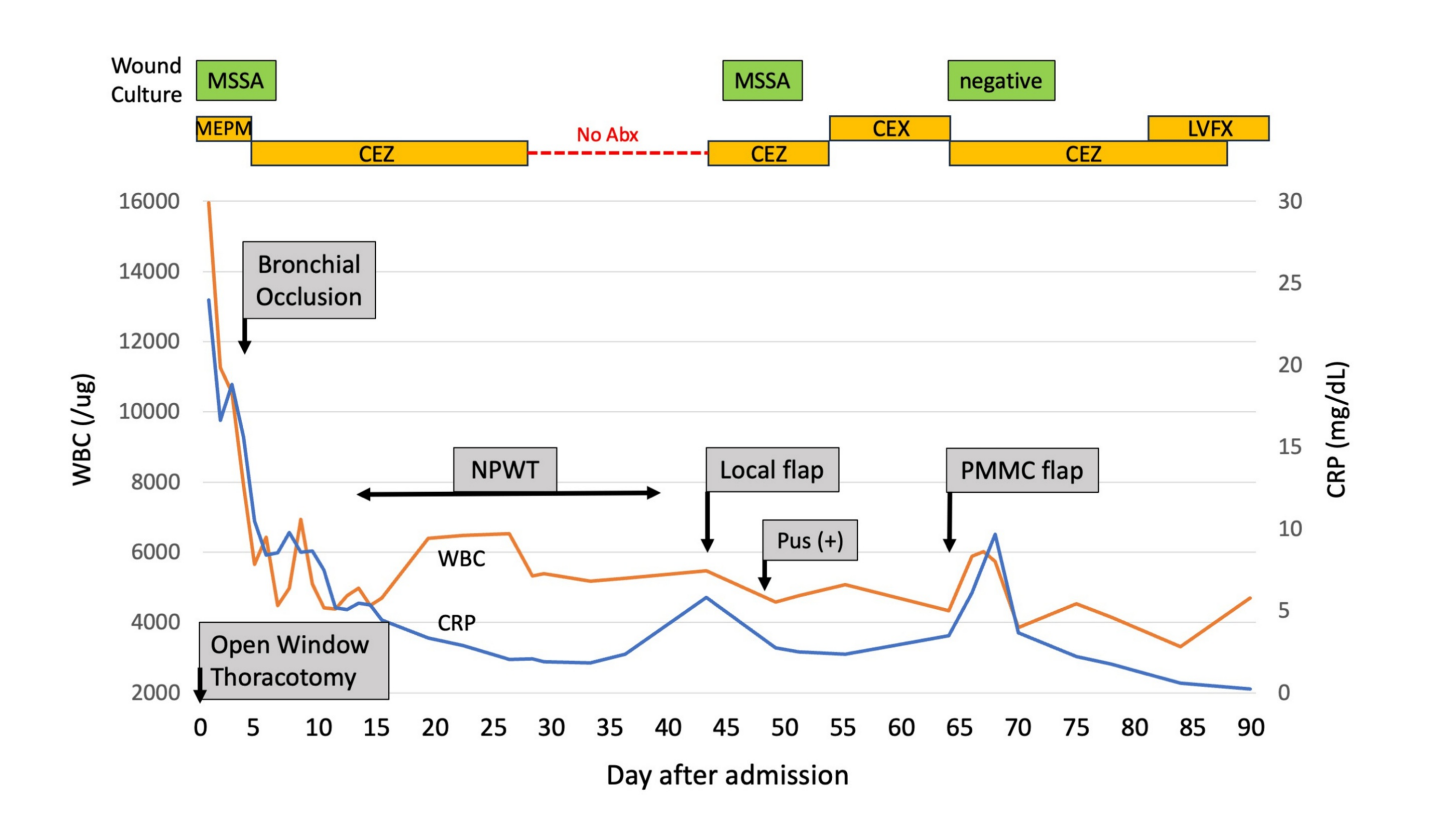
Clinical course MSSA: meticillin-susceptible *Staphylococcus aureus*; Abx: antibiotic; MEPM: meropenem; CEZ: cefazolin; CEX: ceftriaxone; LVFX: levofloxacin; NPWT: negative-pressure wound therapy; PMMC flap: pectoralis major musculocutaneous flap

Initial antimicrobial therapy with meropenem (MEPM) was initiated, and methicillin-sensitive *Staphylococcus aureus* (MSSA) was subsequently identified from blood, sputum, and wound cultures on day four, prompting a switch to cefazolin (CEZ). Endoscopic bronchial occlusion using Endobronchial Watanabe Spigots (EWS) was performed on day two to manage the bronchopleural fistula, followed by continuous wound irrigation. Negative-pressure wound therapy (NPWT) was applied to the wound from day 12 to day 39, leading to the closure of the bronchopleural fistula and favorable granulation formation (Figure 2).

**Figure 3:**
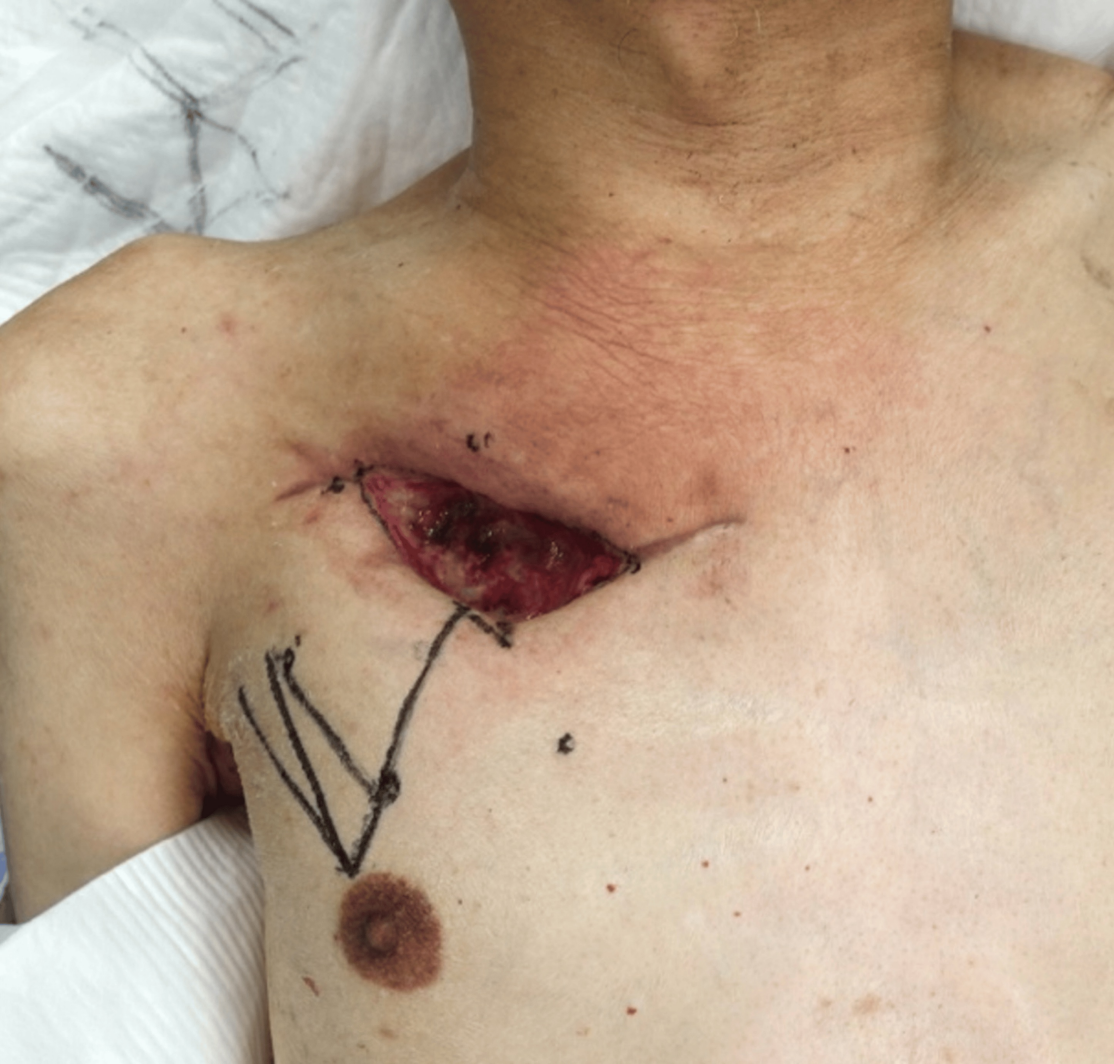
Preoperative appearance of local flap surgery The bronchopleural fistula was closed, and the wound bed seemed to be in good condition.

**Figure 4:**
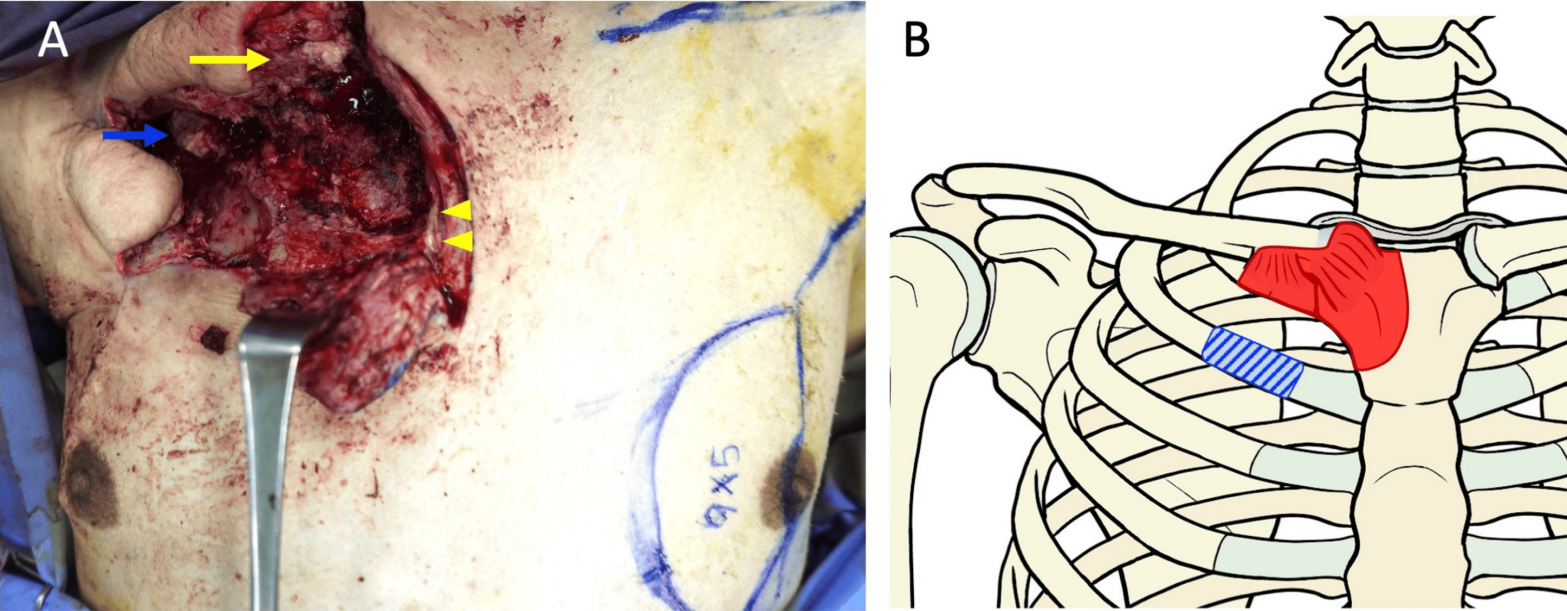
Intraoperative view of pectoralis major myocutaneous flap surgery (A) After resection of the right half of the manubrium sternum, sternoclavicular joint, and first costal cartilage, the left pectoralis major myocutaneous flap was used to fill the defect: debrided clavicle (yellow arrow); resected first rib stump (blue arrow); sternum (yellow arrowheads). (B) Schematic diagram of the resected area: The red hatched area represents the resection range, and the blue diagonally striped area indicates the second rib resected during the initial open-window thoracotomy. Image Credits: Hideyoshi Sato

On day 45, the wound was closed using a local flap (Figure 3), but postoperative wound dehiscence and purulent discharge prompted further intervention. Based on the patient's history and CT findings, residual osteomyelitis associated with SASCJ was suspected. On day 66, the patient underwent resection of the right first costal cartilage, proximal and distal clavicle, and the right half of the manubrium sterni, followed by reconstruction with a left pectoralis major muscle flap (Figure 4).

**Figure 5:**
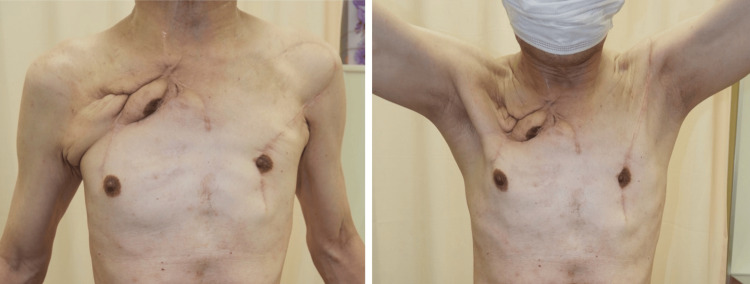
Postoperative view at one year after surgery No recurrence of infection was observed, and there were no restrictions on shoulder joint mobility.

Partial necrosis of the muscle flap occurred postoperatively but was successfully managed conservatively, allowing for discharge on day 92. No recurrence was observed one year postoperatively (Figure 5).

## Discussion

SASCJ is a rare condition that is often treated symptomatically without an accurate diagnosis. In this case, while the treatment initially focused on the fistulous empyema, SASCJ was finally identified after local flap closure. Early diagnosis could have potentially shortened the treatment period, emphasizing the importance of recognizing this condition.

Due to its nonspecific symptoms and gradual progression, many patients present with severe complications, such as mediastinitis or bacteremia, by the time of diagnosis. In this case, the patient sought medical attention four weeks after symptom onset, with progression to empyema accompanied by a bronchopleural fistula. To the best of our knowledge, no reports exist of SASCJ cases complicated by fistulous empyema, and such a severe case requires multiple specialized treatments, including open-window thoracostomy, bronchial occlusion, negative-pressure wound therapy (NPWT), and musculocutaneous flap reconstruction.

Endoscopic bronchial occlusion with EWS has been proven effective for managing persistent air leaks, fistulous empyema, and postoperative bronchial fistulas [7]. In such a case of bronchopleural fistulae with exogenous empyema caused by SASCJ, the responsible bronchus is typically located peripherally and can be clearly identified. Therefore, endoscopic bronchial occlusion using an EWS is considered a suitable treatment option. Closure of the bronchopleural fistula enabled adequate irrigation and decontamination of the wound, which was followed by NPWT to promote reduction of the pleural cavity and pleural adhesion. A combination therapy of endoscopic bronchial occlusion and NPWT was recently reported as one of the effective treatments for fistulous empyema [8,9]. It also proved effective in treating bronchopleural fistula associated with SASCJ, as demonstrated in the present case.

Treatment strategies for SASCJ should be tailored to the disease severity. It includes antibiotic therapy, CT-guided drainage, surgical incision and drainage, and debridement, including SCJ resection. Evaluating the presence of osteomyelitis using MRI is essential [10], and SCJ resection is recommended if osteomyelitis or joint destruction is present [11]. Although some reports indicate that conservative treatment can lead to remission even in cases of osteomyelitis [6,12,13], Song et al. reported recurrences in five out of six cases treated with incision, drainage, and antibiotics alone, underscoring the importance of SCJ resection [14]. Considering reports of recurrence due to insufficient debridement [4,14], aggressive SCJ resection should be considered when there is diagnostic uncertainty.

Functional impairment following SCJ resection is reportedly minimal in most cases [3,14,15]. Ross and Shamsuddin found no movement restrictions in cases involving resection of the medial one-third of the clavicle, one-half of the manubrium, and the first and second costal cartilages [3]. Song et al. [14] recommended limiting manubrium resection to one-half to maintain SCJ stability, while Joethy et al. noted that preserving both pectoralis major muscles prevented shoulder dysfunction, even with complete manubrium resection [16]. However, in three cases with extensive clavicular defects, abduction limitations were observed. Based on these findings, limiting manubrium resection to one-half and clavicular resection to the medial one-third is generally recommended [1,17].

In cases requiring resection of the SCJ and ribs, Ali et al. reported lower recurrence rates in patients undergoing musculocutaneous flap reconstruction compared to those treated with vacuum-assisted closure therapy [2].

Pedicled flaps commonly used in the SCJ region include the latissimus dorsi and pectoralis major flaps. The pectoralis major flap is typically the first choice due to its suitability without the need for patient repositioning during surgery [14]. In this case, the ipsilateral thoracoacromial artery was severed during the initial window thoracostomy, necessitating the use of the contralateral pectoralis major flap. However, the contralateral flap has a limited reach compared to the ipsilateral one, highlighting the importance of preserving the thoracoacromial artery during initial drainage procedures.

## Conclusions

We encountered a rare case of SASCJ that progressed to fistulous empyema. Following a window thoracostomy, endoscopic bronchial occlusion successfully resolved the bronchopleural fistula. In addition, multidisciplinary treatment, including negative-pressure wound therapy, SCJ resection, and reconstruction using a contralateral pectoralis major flap, led to complete recovery.

In severe and advanced cases, collaborative management involving multiple specialties is essential. However, SASCJ remains a rare condition that is sometimes treated symptomatically without a definitive diagnosis. It is crucial for physicians across relevant specialties to be aware of this disease and to establish an appropriate treatment plan to ensure optimal outcomes.
